# Immunodeficiency Promotes Adaptive Alterations of Host Gut Microbiome: An Observational Metagenomic Study in Mice

**DOI:** 10.3389/fmicb.2019.02415

**Published:** 2019-11-01

**Authors:** Shuyu Zheng, Tingting Zhao, Shuijuan Yuan, Lingyu Yang, Jinmei Ding, Li Cui, Mingqing Xu

**Affiliations:** ^1^Shanghai Key Laboratory of Veterinary Biotechnology, School of Agriculture and Biology, Shanghai Jiao Tong University, Shanghai, China; ^2^School of Medicine, Shanghai Jiao Tong University, Shanghai, China; ^3^Bio-X Institutes, Key Laboratory for the Genetics of Developmental and Neuropsychiatric Disorders (Ministry of Education), Shanghai Jiao Tong University, Shanghai, China

**Keywords:** gut microbiota, immunodeficiency, metagenomics analysis, IgA, dysbiosis

## Abstract

The crosstalk between the gut microbiota and immune state of the host is an essential focus in academia and clinics. To explore the dynamic role of the microbiota in response to immune deficiency, we comprehensively assessed the microbiome of 90 mouse fecal samples, across three time points including two immunodeficiency models, namely severe combined immunodeficient (SCID) mice and non-obese diabetic SCID (NOD/SCID) mice, with BALB/cA as a control strain. Metagenomic analysis revealed a decrease in alpha diversity and the existence of a clear structural separation in the microbiota of immunodeficient mice. Although nuances exist between SCID and NOD/SCID mice, an increase in the protective microbiota, in particular *Lactobacillus*, contributed the most to the discrimination of immunodeficient and control mice. Further data regarding the red blood cell (RBC) concentration and serum IgA level during different stages of development support the concept of the microbiota alleviating the advancement of immune deficiency, which is called microbial compensation. Taken together, these results demonstrate the dynamic impact of immunodeficiency on the gut microbiota and the adaptive alteration of the microbiota that may influence the host state.

## Introduction

Multicellular organisms exist as meta-organisms comprising both the macroscopic host and numerous commensal microbiota (Ley et al., 2006). The field of immunology has been transformed by the growing understanding of the crosstalk between the microbiota and the immune system, and the role of the microbiota in the induction, maturation, and function of the immune system along with the effect of shaping and preserving the ecology of the microbiota that the immune barrier imposes (Belkaid and Naik, 2013; Belkaid and Harrison, 2017). The intestinal immune system, unlike any other organ system, is continuously confronted with an enormous microbial load, and thus, a unique adaptation has been developed that allows the immune system to limit opportunistic invasion by the resident microbiota while appropriately responding to bacterial pathogens.

Numerous recent studies highlight the role of the microbiota composition in initiating and perpetuating acute and chronic intestinal inflammation in mice and humans, which has been linked to multifactorial human diseases, such as colorectal cancer and diabetes (Zackular et al., 2014; Levy et al., 2017). Dysbiosis, including bloom of pathobionts, loss of commensals, and loss of diversity, could occur when comparing the data derived from immune disorders such as common variable immune deficiencies to the control (Jørgensen et al., 2016; Berbers et al., 2017). The effects of changes in the intestinal microbiota on immune response and immune cell populations were recently investigated by comparing antibiotic-treated or specific microbe-transplant mice to the wild type (Kawamoto et al., 2014). Conversely, immune dysregulation in primary immunodeficiencies is linked to microbiome dysbiosis (Pellicciotta et al., 2019). However, the current understanding of the interplay between systemic immune response, especially in the context of combined immunodeficiency, and the microbiota remains limited.

Here, the microbiome of 90 mouse fecal samples, across three time points were assessed. A metagenomic analysis of the intestinal microbiota and physiological measurements of relative immune indicators, cells, and organs were conducted. Findings of our study may provide a reference of the role of the microbiota in response to immune deficiency in mice.

## Materials and Methods

### Mice

Total thirty healthy wild-type BALB/cA mice (9–10 g, 3 weeks old) and 60 immunodeficient mice including 30 SCID and 30 NOD/SCID mice were purchased from Shanghai Lingchang BioScience Co., Ltd. (Shanghai, China). Mice were fed with an antibiotic-free combination of 24.5–25.5% crude protein, 5.5–6.5% crude fat, 6.0–6.5% coarse ash, 4.0–4.5% crude fiber, ≤10% water, 1.2–1.5% calcium, 0.75–1.0% phosphorus, ≥1.32% lysine, and ≥0.78% methionine with cysteine. In each isolation room, 2–4 sentinel mice with the same gender were monitored for specific pathogens. Fresh fecal samples (<1 h) were collected from mice and stored at 4°C within 24 h for microbiota genome DNA extraction, or they were stored at –80°C until use. Feces from 10 mice for each strain, consisting of half males and half females, were collected at 4, 8, and 12 weeks, respectively. Animal housing and sacrificing was in accordance with guidelines of Shanghai Jiao Tong University. All experiments involving mice were approved by Animal Ethical Committee of Shanghai Jiao Tong University prior to beginning of the experiment.

### Microbiota Sequencing and Data Processing

Microbiota genome DNA extraction, and 16S rRNA amplification and sequencing were performed following the description given in our previous report (Zhao et al., 2013). Microbial genome DNA was extracted from fecal samples using a QIAamp DNA stool mini kit (QIAGEN, #51504) following the manufacturer’s recommendation. Harvested DNA was measured with a NanoDrop Lite spectrophotometer to estimate DNA quantity and quality. V4 of 16S rRNA was PCR-amplified from microbial genome DNA harvested from fecal samples using barcoded fusion primers (forward primer: 5′-AYTGGGYDTA AAGNG-3′, reverse primer: 5′-TACNVGGGTATCTAATCC-3′), and was used for the remainder of our study. The PCR conditions and PCR product purification followed procedures that are described in our previous publication (Zhao et al., 2013). The purified barcoded V4 amplicons were sequenced using the pair-end method and the Illumina MiSeq platform. Sequencing was carried out by the Hangzhou Hegu Biotechnology Co., Ltd. The DNA sequences are publicly available in Metagenomic Rapid Annotation using Subsystem Technology (MG-RAST) under the project name “immunodeficient_microbiota.” Sequences with an average phred score lower than 25, containing ambiguous bases, a homopolymer run exceeding 6, having mismatches in primers, or sequence lengths shorter than 100 bp were removed. Only sequences with an overlap longer than 10 bp and without any mismatches were assembled according to their overlap sequence. Sequences that could not be assembled were discarded. Barcodes and sequencing primers were trimmed from the assembled sequence (Supplementary Figure S1). Trimmed sequences were uploaded to Quantitative Insights into Microbial Ecology (QIIME) for further examination with its software for microbial community analysis.

### Taxonomy Classification and Analysis

The trimmed and assembled sequences from each sample were aligned to Greengene 16S rRNA training set 10 using the best hit option to classify the taxonomy abundance in QIIME^1^. Bacterial operational taxonomic units (OTUs) were generated using the Uclust algorithm function in QIIME^2^ (Caporaso et al., 2010). Permutational multivariate analysis of variance using distance matrices (ADONIS) and analysis of similarity (ANOSIM) were analyzed by QIIME. ACE, Chao, and the Simpson and Shannon index were calculated using Mothur software. The linear discriminant analysis (LDA) effect size (LefSe) method was applied to identify microbes of different taxa among lines using the default parameters (LDA Score > 2, *p* < 0.05) (Segata et al., 2011).

### Functional Predictions of Gut Microbiota

Microbial functions were predicted using Phylogenetic Investigation of Communities by Reconstruction of Unobserved States (PICRUSt) software (Langille et al., 2013). The OTUs were mapped to the gg13.5 database at 97% similarity by QIIME’s command “pick_closed_otus.” The OTU abundance was automatically normalized using 16S rRNA gene copy numbers from known bacterial genomes in Integrated Microbial Genomes (IMG). The predicted genes and their functions were aligned to the Kyoto Encyclopedia of Genes and Genomes (KEGG) database, and differences among groups were compared using STAMP software^3^ (Parks and Beiko, 2010). A two-sided Welch’s *t*-test and the Benjamin–Hochberg false discovery rate (FDR) (*p* < 0.05) correction were used in two-group analyses.

### Physiological Index Measurements

Each group of 20 mice with half females and half males aged among 4, 8, and 16 weeks was euthanized. Samples were collected immediately after euthanizing the mice. Organs including the heart, liver, spleen, lung, kidney, adrenal gland, thymus, brain, and testis/ovary were weighed. Indicators related to blood cells, metabolic enzymes in whole blood, and classes of serum immunoglobulins including IgG, IgA, and complements C3 and C4 were analyzed with a Sysmex KX-21N automatic blood cell analyzer (Hitachi, Japan). Additionally, T, B(B220 +), and NK cells were measured by flow cytometry (BD Biosciences, United States).

### Statistics

Statistical analysis was performed by SPSS version 22.0 (SPSS Inc., Chicago, IL, United States) Group measures are expressed as mean + SEM. Statistical significance was tested using analysis of variance (ANOVA) to compare more groups, with Fisher’s Least Significant Difference (LSD) Test for multiple testing. Significance is indicated on figures as follows: N.S. (not significant), ^∗^*p* < 0.05, ^∗∗^*p* < 0.01, ^∗∗∗^*p* < 0.001, ^∗∗∗∗^*p* < 0.0001.

## Results

### Reduced Diversity and Shifted Composition of the Gut Microbiota in Immunodeficient Mice

The gut microbiota of 90 samples, including two lines of immunodeficient mice, SCID and NOD/SCID mice, and one physiologically normal line BALB/cA (Supplementary Additional File S1), was profiled by collecting fecal samples at 4, 8, and 12 weeks and conducting 16S rRNA amplicon sequencing. The microbial classifications revealed that despite sex and age, on average, 34 phyla, 58 orders, and 254 genera were present in BALB/cA mice; 33 phyla, 44 orders, and 222 genera were present in SCID mice; and 25 phyla, 41 orders, and 226 genera were present in NOD/SCID mice (Supplementary Additional File S2).

**FIGURE 1:**
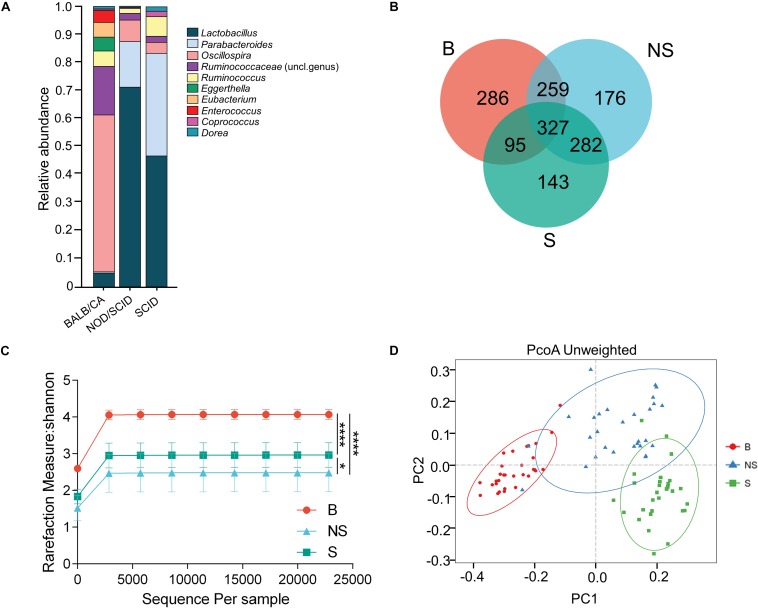
Compositional separation of the microbiota in three different groups. **(A)** Genus-level microbial classification of bacteria from BALB/cA, NOD/SCID, and SCID mouse stool samples. **(B)** Venn diagram of OTUs of the fecal microbiota in three groups. **(C)** Diversity of bacterial species (>97% identity), as indicated by the Shannon rarefaction measure. **(D)** Unweighted Unifrac metrics separate the BALB/cA mice (red dot), NOD/SCID mice (blue triangle), and SCID mice (green square) microbiota. Each symbol represents a sample. B, BALB/cA mice; NS, NOD/SCID mice; S, SCID mice; ^∗^*p* < 0.05, ^∗∗∗∗^*p* < 0.0001.

A clear distinction between immunodeficient mice and BALB/cA mice could be observed at the genus level, with SCID and NOD/SCID mice being dominated by *Lactobacillus* (71.3 and 46.7%, respectively), followed by *Parabacteroides* (16.7 and 37.2%), and BALB/cA mice by *Oscillospira* (57.1%), followed by *Ruminococcaceae* (17.2%) (Figure 1A). Additionally, *Enterococcus*, *Eubacterium*, and *Eggerthella* were high in BALB/cA mice while low in SCID and NOD/SCID mice. In addition, the NOD/SCID mouse group presented lower microbial diversity than the SCID mouse group (*p* < 0.05, Figure 1A).

Operational taxonomic unit analysis showed that a total of 967, 1044, and 847 OTUs were obtained from BALB/cA, NOD/SCID, and SCID mouse stool samples, respectively (Figure 1C). There were 327 common OTUs in the three groups (Figure 1B). A significant reduction in alpha diversity was observed in the two immunodeficient mouse groups, compared to the BALB/cA mouse group, as estimated by the Shannon indices (*p* < 0.0001, Figure 1C).

Multivariate statistical analyses were performed to compare the overall structure of the gut microbiota of all samples. Principle coordinates analysis (PCoA, Figure 1D) based on the relative abundance of OTUs revealed a clear separation among the three groups, on the basis of the first two principal component scores up to 32.14 and 14.33%. A multivariate ANOVA test further confirmed that the immune condition was a dominant source of variation (*p* < 0.05), rather than sex or age (age groups of 4, 8, and 12 weeks). Consistent results were obtained by non-metric multidimensional scaling (NMDS, Supplementary Figure S1A) and partial least squares discrimination analysis (PLS-DA) analyses (Supplementary Additional File S4).

Additionally, three alpha diversity index, chao1 index, Shannon index, Simpson index, and ace index in the three mouse groups were analyzed (Supplementary Additional File S3 and Supplementary Figure S2). The Shannon index and Simpson index in the BALB/cA mouse group were significantly higher than those in the NOD/SCID mouse group and SCID mouse group (^∗∗∗^*p* < 0.001 or ^∗∗∗∗^*p* < 0.0001).

### Microbiota Are Associated With Immune Deficiency

To further identify specific bacterial taxa associated with the immune state, we compared the fecal microbiota among the three groups using the linear discriminate analysis (LDA) effect size (LEfSe) method. A significant shift of the microbiota based on relative abundance is illustrated in a cladogram among the three groups (Supplementary Figure S3A). The abundance of the shared 11 genera was higher in the BALB/CA mouse group compared with the other two groups. Additionally, *Parabacteroides* and butyrate-producing bacteria *Roseburia* was higher in the SCID group compared with the BALB/CA group, along with a significantly higher distribution of the acetate-producing bacteria *Blautia* in the NOD/SCID group than that in the BALB/CA group (Supplementary Figure S3B).

**FIGURE 2:**
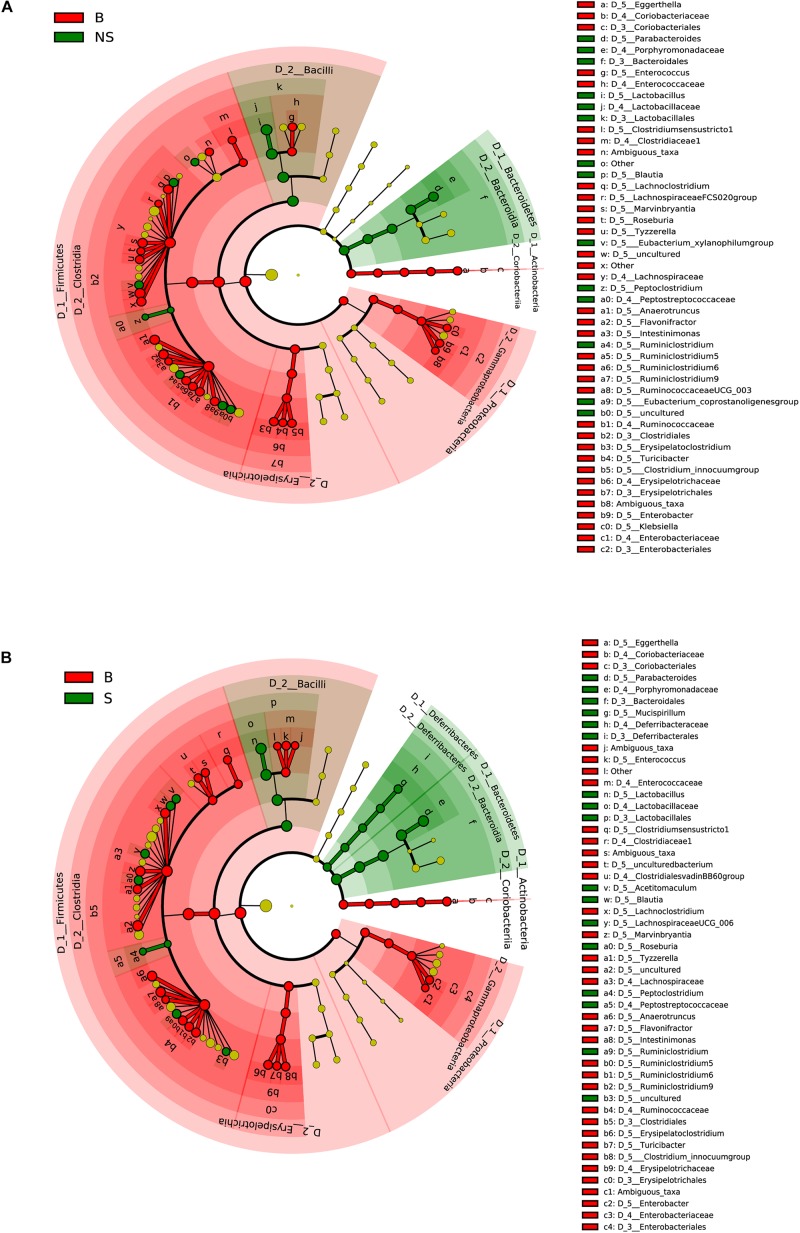
Different structures of the microbiota in BALB/cA and NOD/SCID mice or BALB/cA and SCID mice. **(A)** Taxonomic representation of statistically and biologically consistent differences between BALB/cA and NOD/SCID mice. Differences are represented by the color of the most abundant class (red indicates BALB/cA mice, and green indicates NOD/SCID mice). **(B)** Taxonomic representation of statistically and biologically consistent differences between BALB/cA and SCID mice. Differences are represented by the color of the most abundant class (red indicates BALB/cA mice, and green indicates SCID mice). The diameter of each circle is proportional to the taxon’s abundance.

**FIGURE 3:**
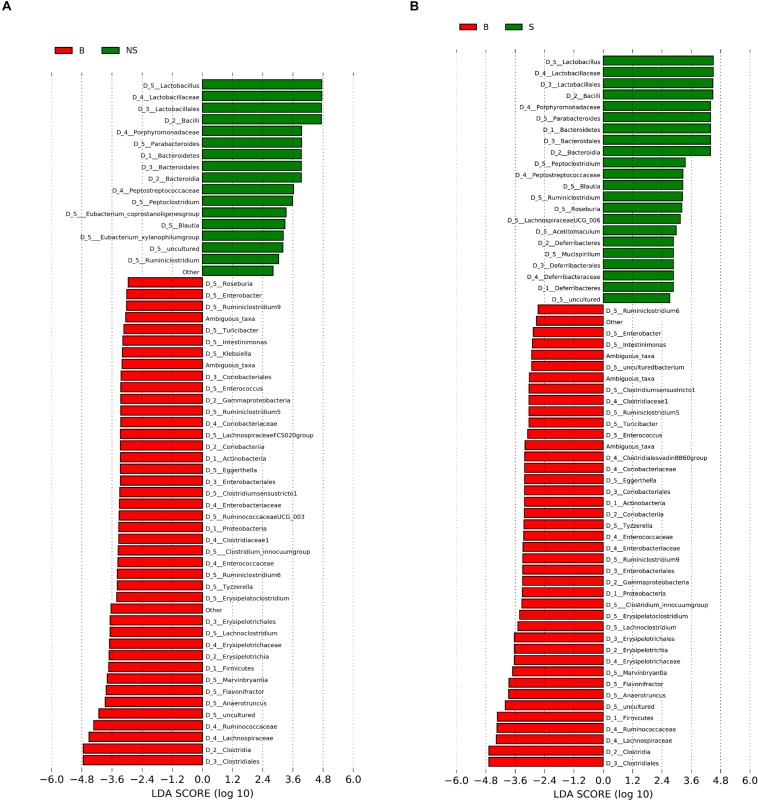
Histogram of the LDA scores for differentially abundant genera between **(A)** BALB/cA and NOD/SCID mice or **(B)** BALB/cA and SCID mice. The cladogram was calculated by LEfSe, a metagenome analysis of abundant taxons of OTUs; the cladogram is displayed according to the effect size. Only taxa meeting an LDA significant threshold > 2 and *p* < 0.05 are shown.

When conducting a paired comparison, the number of taxa preceding the LDA threshold was larger in BALB/cA group than that in the NOD/SCID group. At the phylum level, the abundance of *Bacteroidetes* was higher in the NOD/SCID group compared to BALB/cA mice, while that of *Actinobacteria*, *Firmicutes*, and *Proteobacteria* was higher in BALB/cA mice (Figure 2A). At the level of class, *Bacilli* and *Bacteroidia* increased in NOD/SCID mice. The numbers of genera that differed for BALB/cA vs. NOD/SCID (Figure 2A) and BALB/cA vs. the SCID group (Figure 2B) were 28 and 26, respectively. Among genera, all 14 belonging to *Clostridia* (LDA score = 4.78) were decreased in NOD/SCID compared to BALB/cA, and for SCID mice, the respective number was 15 (Figure 3A). The sharpest increase in genera occurred for a *Lactobacillus* probiotic in the NOD/SCID and SCID groups (LDA score = 4.78 and 4.62, respectively), compared to the BALB/cA group (Figure 3B). Interestingly, compared to BALB/cA, several mutual genera including the short-chain fatty acid (SCFA)-producing bacteria *Parabacteroides*, *Lactobacillus*, *Blautia*, *Peptoclostridium*, and *Ruminiclostridium* with the capability of fiber degradation were both enriched in the NOD/SCID group and SCID group.

### Functional Pathways of the Microbiota Engaged in Immune Deficiency

**FIGURE 4:**
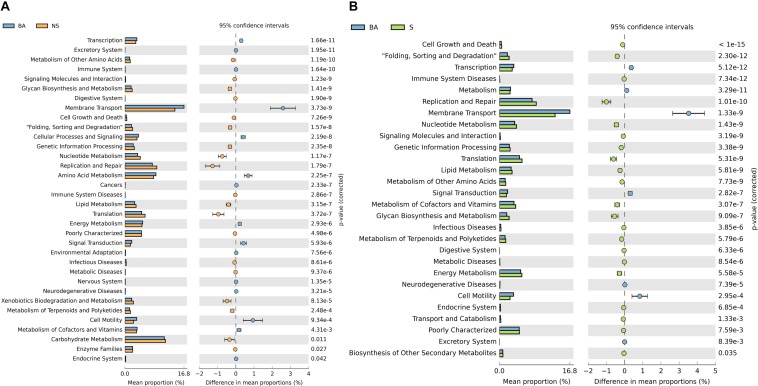
Prediction of bacterial metabolic function. Comparisons of KEGG pathways predicted using PICRUSt between microbes of BALB/cA mice and NOD/SCID mice **(A)** and BALB/cA mice and SCID mice **(B)**.

To better understand the role of the microbiota in immune deficiency-related disease, we then conducted functional pathway analyses based on the KEGG database. A discrepancy in pathways relating to nutrient metabolism on KEGG level 2 was observed when comparing the BALB/cA group to two immune deficiency groups (Figures 4A,B and Supplementary Additional File S5). Amino acid metabolism was significantly activated in the BALB/cA group, while glycan biosynthesis and metabolism, nucleotide metabolism, lipid metabolism, and metabolism of terpenoids and polyketides were silenced compared to the SCID and NOD/SCID groups (*p* < 0.05).

### Dynamic Profiles of the Microbiota

**FIGURE 5:**
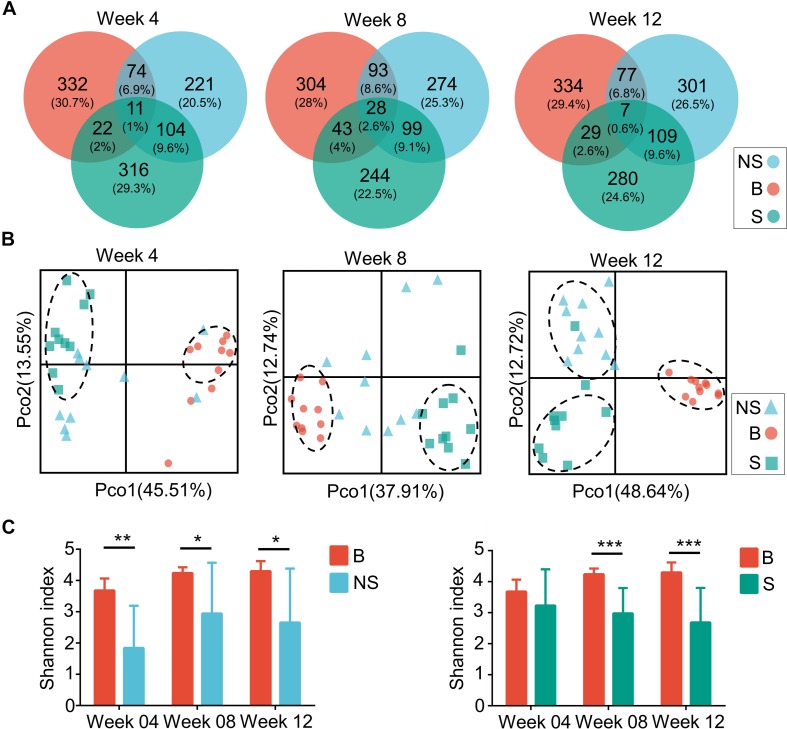
Microbiota compositions of three groups during several weeks. **(A)** Venn diagrams of OTUs of the fecal microbiota in BALB/cA (B), NOD/SCID (NS), and SCID (S) mouse. **(B)** PCoA plots based on weighted Unifrac metrics. Each symbol represents a sample. **(C)** Alpha diversity plots of the fecal microbiota of the three groups over time. ^∗^*p* < 0.05, ^∗∗^*p* < 0.01, ^∗∗∗^*p* < 0.001.

As aforementioned, a decrease in diversity, and a rescue shift in the microbial community and functional pathways predisposed to immune disease were shown in the immunodeficient groups compared to the control. In order to elucidate the influence of the host’s physical background on gut microbiota, we then studied the microbial profile during different stages. Throughout week 4 and 12, the shared OTUs detected from the fecal microbiota of the three groups increased from 11 to 28 (1 and 2.6% of the total, respectively), and then fell to 7 (0.6% of the total) (Figure 5A). Simultaneously, a stepwise decrease in the relative number of OTUs of the NOD/SCID group that overlapped with the other two groups was observed, while that of the SCID group was increased on the whole.

**FIGURE 6:**
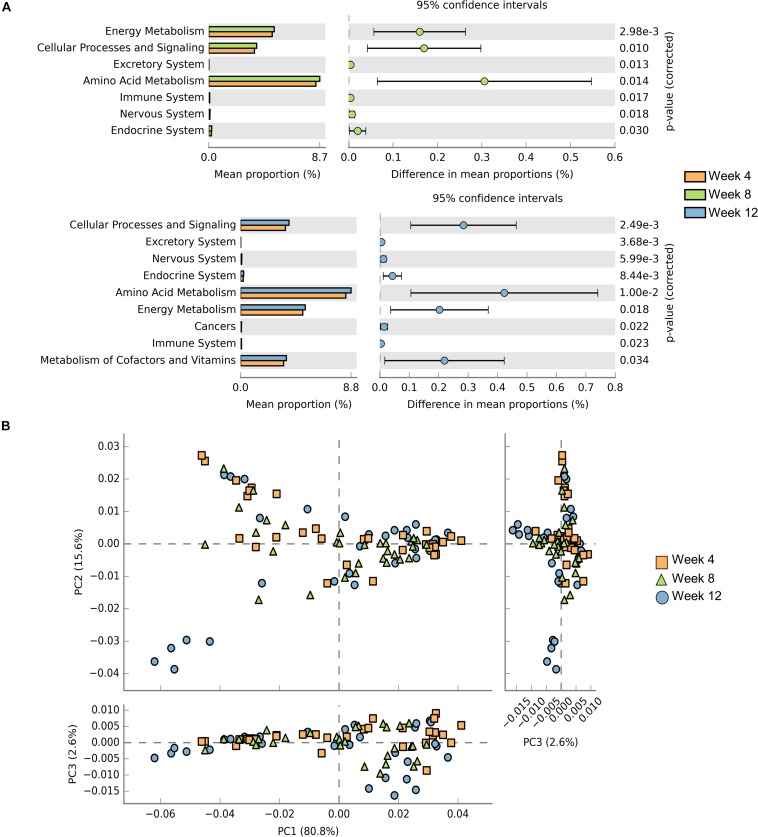
Comparisons of functional pathways among microbes for three endpoints. **(A)** Comparison of the functional pathway of KEGG between microbes collected from mice at week 4 (orange), week 8 (green), and week 12 (blue). **(B)** PCA plots of microbes collected from week 4 (orange rectangle), week 8 (green triangle), and week 12 (blue circle) mice. Only microbes meeting *p* < 0.05 are shown.

Samples contribution rates of the first PCoA (PC1) at week 4, 8, and 12 were 13.55, 12.74, and 12.72%, respectively, which displayed a clear separation of the SCID group, NOD/SCID group and BALB/cA group (Figure 5B). Similarly, alpha diversity with the Shannon index was decreased in NOD/SCID group and SCID group compared with the BALB/cA group in week 8 and 12 (Figure 5C). Specifically, the functional pathway comparison showed that the pathways involving energy metabolism, cellular processes and signaling, and amino acid metabolism were significantly enriched in week 8 and week 12 mice, compared to week 4 mice (*p* < 0.05) (Figure 6A), and a dispersed trend could be seen in the week 12 mouse group from the principal component analysis (PCA) (Figure 6B), when considering age as the first variable (with the first two principle scores of 80.8 and 15.6%).

### A Rescue Phenomenon Is Associated With the Microbiota

**FIGURE 7:**
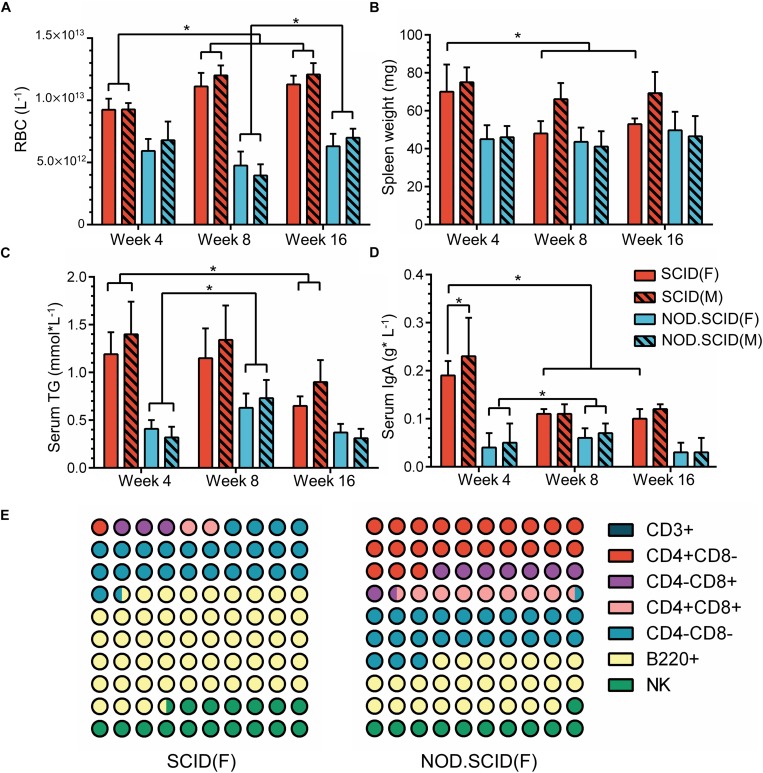
Physiological variations of immunodeficient mice. **(A)** Concentration of RBCs (L^–1^) in whole blood among 4-, 8-, and 16-week immunodeficient mice. **(B)** Spleen weights (mg) among 4-, 8-, and 16-week immunodeficient mice. **(C)** Concentration of serum triglyceride (TG) (mmol × L^–1^) among 4-, 8-, and 16-week immunodeficient mice. **(D)** Levels of serum IgA (g × L^–1^) among 4-, 8- and 16-week immunodeficient mice. **(E)** Composition of immune cells among 8-week female immunodeficient mice. The male mice show a similar trend as corresponding female mice. For detailed information, please refer to Supplementary Additional File S6. The total part is 100%; ^∗^*p* < 0.05.

The above results further drove us to examine the physiological index that relates to the host immune state. A total of 120 mice including week 4, 8, and 16 (40 at each time point) were collected (Supplementary Additional File S6). Blood physiological tests exhibited an overall decrease in red blood cell (RBC) concentration, with an average of 10.83 × 10^12^ and 5.79 × 10^12^ L^–1^ in SCID and NOD/SCID mice, respectively, which was much lower than that of BALB/cA mice. However, with no difference between sexes, the concentration of RBCs increased in week 8 and 16 SCID mice compared to the week 4 group (*p* < 0.05), and that increase could also be observed in week 16 NOD/SCID mice compared to the week 8 group (*p* < 0.05, Figure 7A).

Additionally, compared to BALB/cA mice in week 4, a continued weight reduction in the spleen, and weight increasion in the heart, lung, liver, brain and kidney were observed in the female immunodeficient mice in week 8 and 16 (Figure 7B and Supplementary Additional File S6). Moreover, serum triglyceride (TG), which is biochemical indicator relative to metabolism, exhibited lower concentrations in SCID and BALB/cA mice, but was increased in the week 8 in NOD/SCID group (*p* < 0.05) (Figure 7C and Supplementary Additional File S6).

Among detected serum immunoglobulins, the concentration of IgA was significantly decreased in week 8 and week 16 female SCID mice, compared to the same sex mice of week 4 (*p* < 0.05) (Figure 7D and Supplementary Additional File S6). For NOD/SCID mice, the serum IgA initially increased from 0.045 to 0.065 g × L^–1^ on average (*p* < 0.05). In addition, different compositions of immune cells were observed, with dominant B220^+^ cells in SCID female mice, and a smaller share in NOD/SCID female mice. The relative amounts of CD4^+^CD8^–^ T and CD4^+^CD8^+^ T cells were large in the NOD/SCID group, but were rare (3.13 ± 2.41 and 2.02 ± 1.10%, respectively) in the SCID group, presenting a different immune pattern between two mouse lines (Figure 7E and Supplementary Additional File S6).

## Discussion

Although rapid progress has been made regarding our understanding of the relationship between innate and adaptive immunity and the gut microbiota (Honda and Littman, 2016; Thaiss et al., 2016), little is known regarding the impact of immune deficiency on the microbiota and the adaptation of the microbiota under such circumstances. Here, we used SCID mice characterized by combined deficiency in T and B cells, and NOD/SCID with innate and adaptive immune deficiency to expand our understanding of the interaction between the host immune system and the gut microbiome. Additionally, we detected the microbiome as well as the physiological index relative to immunity and metabolism at three different endpoints.

In the current study, an obvious decrease in alpha diversity was found in the immunodeficient group, suggesting that this is a consequence of microbial dysbiosis, which may therefore predispose the mice to inflammatory complications and infections as previously reported (Nagalingam et al., 2013; Ray and Dittel, 2015; Kishida et al., 2018). We also found a clear separation among the three groups in the overall OTU structure, which was positively related to time, and noted the increased bacteria including SCFA-producing genera as well as fiber-degradation genera that distinguished the immunodeficient mice from the control. Notably, the dominant species in the bacterial community in immune-deficient mice was *Lactobacillus*, which was significantly more abundant compared to the control. More interestingly, an overall decrease in the concentration of serum immunoglobulins, RBCs, and weights of spleen were observed with increasing age.

In this study, a reduction in alpha diversity among the SCID and NOD/SCID mouse groups was observed. There is a precedent in the literature supporting the role of gut microbiota in immunodeficient diseases, including clinical observation and lab-controlled confirmation. A decrease in bacterial diversity and levels of IgA along with increased dysbiosis were observed in common variable immune deficiency (CVID) patients (Jørgensen et al., 2016). Dysbiosis in the gut microbiota was also present in other immunodeficiencies such as those involving interleukin-10, -17, or -23 (Cua and Tato, 2010; Godinez et al., 2011; Nagalingam et al., 2013; Schurich et al., 2017), nucleotide-binding oligomerization domain (NOD)-like receptors (NLR) (Frank et al., 2011; Rehman et al., 2011; Arai et al., 2015; Hu et al., 2018), or toll-like receptors (Carvalho et al., 2012; Ubeda et al., 2012), and the susceptibility to colitis observed in the corresponding immunodeficient mice showed a clear dependence upon the composition of the microbiome.

Innate immunity plays an important role in gut homeostasis, which builds bridges between luminal and internal environments in order to successfully interact with adaptive immunity factors to recognize pathogenic bacteria while tolerating commensals. The much lower levels of classes of serum immunoglobulins and the biochemical index as well as the lesser weights of immune organs in NOD/SCID mice indicated the importance of innate immunity. As a signaling hub, the intestinal microbiome senses the environmental inputs and translates that signal through metabolism and the innate immune system to respond. The increased metabolic pathways include xenobiotic biodegradation and metabolism, and carbohydrate metabolism in NOD/SCID mice, while that of cofactors and vitamins in SCID mice compared to BALB/cA may provide clues as to how aberrations in microbiota-immune communication would affect metabolism (Sobko et al., 2005, 2006).

Given that T and B cells have pivotal functions for maintaining immune homeostasis through suppressing responses to harmless antigens, the intervention of the differentiation process of immune-reactive cells such as Th17 cells would be of importance to immune tolerance. Studies on segmented filamentous bacteria (SFB) revealed the role of the microbiota in Th17 cell induction, causing autoimmune arthritis in susceptible mice (Ivanov et al., 2009). Accounting for 30–40% of differentiated memory CD4^+^T cells in the lamina propria of the intestine (Ivanov et al., 2008). However, those cells could also trigger an inflammatory response through expressing pro-inflammatory cytokines. The increased proportion of CD4^+^T cells, and a rescue in the serum IgA level in NOD/SCID mice compared to SCID mice may indicate an increased reaction of Th17 cells, although the inner relationship between more enriched genera such as *Lactobacillus* and *Oscillospira* and immune response was unclear.

Consistent with previous studies, mucosal IgA is central in holding the relationship between the immune system and the resident microbiota (Pabst et al., 2016). IgA not only acts as a first line of defense by neutralizing viruses and exotoxins, preventing attachment, and limiting the access of microorganisms to the epithelium (Strugnell and Wijburg, 2010; van de Ven et al., 2014), but it also maintains the interplay between commensals, epithelium, and immune system through imposing selective pressure on the microbial consortium (Macpherson et al., 2015). The absence of hypermutated intestinal IgA in AID^–/–^ mice led to a more anaerobic environment dominated by SFB, and the presence of IgA could reverse the phenomena (Suzuki et al., 2004). We found that in SCID mice, the serum IgA level was high at 4 weeks then declined at 8 weeks. For NOD/SCID mice, although a much lower level of IgA was observed, the trend was not obvious during the progress of immune deficiency. Additionally, there was less decrease in alpha diversity of the microbiota during week 8 and week 16 compared to week 4 in NOD/SCID mice. Additionally, the pathways involving energy metabolism, cellular process and signaling, and amino acid metabolism were more enriched at 8 and 16 weeks. These results depict a synchronous change between the host immune system and the microbiota to a more beneficial state over time.

In the current study, some beneficial microbiota including the SCFA-producing microbiota *Parabacteroides*, *Lactobacillus*, and *Blautia*, and fiber-degradation bacteria *Ruminiclostridium* were significantly more abundant in immunodeficient mice. With the abilities of depolymerization and fermentation of dietary polysaccharides into host-absorbable SCFAs, these SCFA-producing microbiota were found to be positively related to weight loss (Qin et al., 2012) and the protection of the colonic mucus barrier. Of note, the well-known probiotic *Lactobacillus* has been proven to stimulate and enhance the immune system both in mouse experiments and clinical trials (Cross, 2002; Jones, 2017). Additionally, an increased proportion of Bacteroidetes at the phylum level in NOD/SCID mice generally suggests that a beneficial adaptation of the microbiota community occurred regarding their specific enzymes for polysaccharide degradation (Gibiino et al., 2018). Although the inner mechanism requires further investigation, these changes are a collective presentation of compensation for immune barrier deficiency.

This study is not without limitations. Although the association between physiological index relative to immunity and microbial community change was observed, whether the alteration in the microbiota was a cause or result remains unclear. Moreover, fecal microbiota transplantation or other intervention will allow for a more complete investigation of the inner mechanism of compensation. Furthermore, additional data are needed to increase our understanding of the differences upon dynamic physiological and microbial changes between the two immune deficiency strains.

## Conclusion

### Reduced Diversity and Shifted Composition of the Gut Microbiota in Immunodeficient Mice

We demonstrated that a decrease in alpha diversity and a clear structural separation exist in the microbiota of immunodeficient mice. Although nuances exist between SCID and NOD/SCID mice, an increase in the protective microbiota, in particular *Lactobacillus*, contributed the most to the discrimination of immunodeficient and control mice. Further data regarding the RBC concentration and serum IgA level during different stages of development will support the concept of the microbiota alleviating the advancement of immune deficiency, which we call microbial compensation.

## Data Availability Statement

The 16S sequences are publicly available in Metagenomic Rapid Annotation using Subsystem Technology (MG-RAST) under the project name “immunodeficient_microbiota” with project ID mgp89476.

## Ethics Statement

The animal study was reviewed and approved by the Shanghai Jiao Tong University. Written informed consent was obtained from the owners for the participation of their animals in this study.

## Author Contributions

SY was responsible for data collection. SZ and TZ wrote the first draft and revised the manuscript. LY, JD, LC, and MX revised the manuscript. LC and MX were responsible for the conception and design of the manuscript and revised it critically, had full access to all the data in the study, and took responsibility for the integrity of the data and the accuracy of the data analysis. All authors approved the final manuscript.

## Conflict of Interest

The authors declare that the research was conducted in the absence of any commercial or financial relationships that could be construed as a potential conflict of interest.

## Footnotes

1 http://qiime.org/index.html

2 http://qiime.org/scripts/pick_otus.html

3 http://kiwi.cs.dal.ca/Software/STAMP
