# Supplementary material for: Immunodeficiency Promotes Adaptive Alterations of Host Gut Microbiome: An Observational Metagenomic Study in Mice
Source: Front Microbiol. 2019 Nov 1;10:2415. doi: 10.3389/fmicb.2019.02415 (PMC6853035; doi:10.3389/fmicb.2019.02415)
Supplement: Supplementary file 1 [file Table_1.docx]

## Supplementary Additional File 6. Procedures for mice physiological and immunological indexes measurements

### Organ collection and measurements

Methods

Each group of 20 mice with half females and half males aged among 4, 8, and 16 weeks was euthanized. Organs including the heart, liver, spleen, lung, kidney, adrenal gland, thymus, brain, and testis/ovary were immediately collected and were processed with filter paper to suck the moisture of tissue surface. All of the organs were weighted by an electronic balance (Mettler toledo AL104, Shanghai, China). Data were processed by SPSS 19.0 for one-way ANOVA.

Results

Table 1 Organ weights of SCID mice

| Organs (mg) | Week 4 | | Week 8 | | Week 16 | |
| --- | --- | --- | --- | --- | --- | --- |
|  | ♀（*n*=10） | ♂（*n*=10） | ♀（*n*=10） | ♂（*n*=10） | ♀（*n*=10） | ♂（*n*=10） |
| heart | 73.30±8.13 | 79.30±8.69 | 98.40±16.00^#^ | 123.00±14.60^*#^ | 107.27±13.00^#^ | 139.67±19.61^*#^ |
| Lung | 108.00±9.30 | 116.00±17.30 | 127.00±16.80^#^ | 143.00±16.20^*#^ | 148.6±16.74^#^ | 155.07±21.55^#^ |
| Liver | 744.00±99.20 | 855.00±92.00^*^ | 1036.00±88.60^#^ | 1280.00±106.00^*#^ | 1131.07±75.90^#^ | 1376.53±116.77^*#^ |
| Spleen | 70.00±14.40 | 75.00±7.91 | 48.00±6.57^#^ | 66.10±8.51^*^ | 52.93±3.05^#^ | 69.27±11.18^*^ |
| Brain | 334.00±21.20 | 351.00±35.60 | 362.00±27.80^#^ | 352.00±36.50 | 373.87±34.03^#^ | 375.00±25.75 |
| Kidney | 91.50±9.12 | 100.00±10.52^*^ | 125.00±13.6^#^ | 190.50±17.90^*#^ | 140.00±10.72^#^ | 194.90±18.45^*#^ |
| Adrenal | 6.13±1.25 | 5.27±1.71 | 7.53±1.55^#^ | 4.47±1.60^*^ | 8.20±1.42^#^ | 4.60±1.40^*^ |
| Thymus | 16.20±9.30 | 18.80±6.06 | 20.30±10.50 | 20.90±8.84 | 20.93±9.55 | 25.53±17.03 |
| Ovary | 3.50±1.57 |  | 3.83±1.10 |  | 5.65±2.11^#^ |  |
| Testis |  | 32.01±4.94 |  | 66.57±6.08^#^ |  | 70.87±8.87^#^ |

* comparison of different sexes with the same age, p<0.05

# comparison to week 4 mice with the same gender, p<0.05

Table 2 Organ weights of NOD/SCID mice

| Organs (mg) | Week 4 | | Week 8 | | Week 16 | |
| --- | --- | --- | --- | --- | --- | --- |
|  | ♀（*n*=10） | ♂（*n*=10） | ♀（*n*=10） | ♂（*n*=10） | ♀（*n*=10） | ♂（*n*=10） |
| Heart | 67.08±6.73 | 91.41±13.37^*^ | 121.66±14.41^#^ | 138.76±17.63^*#^ | 121.47±19.78^#^ | 152.67±29.35^*#^ |
| Lung | 109.51±10.31 | 119.29±11.20^*^ | 167.97±17.73^#^ | 189.53±13.90^*#^ | 183.97±23.50^#^ | 189.05±26.61^#^ |
| Liver | 593.05±94.87 | 774.00±95.69^*^ | 1116.15±136.88^#^ | 1473.93±118.95^*#^ | 1033.86±123.12^#^ | 1401.28±256.15^*#^ |
| Spleen | 45.05± 7.36 | 46.06±5.90 | 43.59±7.52 | 41.14±8.07 | 49.73±9.73 | 46.55±10.68 |
| Brain | 420.55±16.62 | 425.59±33.50 | 461.71±36.18^#^ | 453.17±25.98^#^ | 479.92±44.59^#^ | 478.82±31.72^#^ |
| Kidney | 174.26±16.29 | 253.58±21.11^*^ | 307.19±22.47^#^ | 486.74±45.29^*#^ | 332.83±81.05^#^ | 521.39±68.93^*#^ |
| Adrenal | 4.26±1.03 | 5.04±0.82^*^ | 6.86±1.60^#^ | 6.07±1.76^#^ | 6.79±1.72^#^ | 4.83±0.97^*#^ |
| Thymus | 8.80±3.06 | 10.40±4.01 | 5.23±2.10 | 6.92±2.33 | 5.03±1.50 | 6.33±1.84 |
| Ovary | 6.60±1.78 |  | 18.44±3.46^#^ |  | 21.55±4.40^#^ |  |
| Testis |  | 60.59±14.77 |  | 159.68±25.67^#^ |  | 171.31±22.53^#^ |

* comparison of different sexes with the same age, p<0.05

# comparison to week 4 mice with the same gender, p<0.05

### Peripheral blood collection and measurements

Methods

Orbital vein blood was collected from each group of 20 mice with half females and half males aged among 4, 8, and 16 weeks. For each mice, 20 µL whole blood was diluted with 500 µL 0.85% saline, with the following automatic blood cell analyse (Sysmex KX-21N Automatic blood cell analyser, Hitachi, Japan). At the same time, 0.8 µL whole blood of each mice was collected in the centrifugal tube, then stored in 4℃ for 1h with the following centrifugation at 2800 x g for 10 min at 4°C( Eppendorf 5427R centrifuge, Hamburg, Germany) to extract serum.

Results

Table 3 Physiological indexes of SCID mice

| Indexes | Week 4 | | Week 8 | | Week 16 | |
| --- | --- | --- | --- | --- | --- | --- |
|  | ♀（*n*=10） | ♂（*n*=10） | ♀（*n*=10） | ♂（*n*=10） | ♀（*n*=10） | ♂（*n*=10） |
| WBC/10^9^·L^-1^ | 2.85±0.93 | 2.71±0.53 | 2.99±0.66 | 3.66±0.68^*#^ | 3.26±0.41^#^ | 4.37±0.63^*#^ |
| RBC/10^12^·L^-1^ | 9.24±0.89 | 9.27±0.51 | 11.12±1.09^#^ | 12.01±0.79^*#^ | 11.28±0.70^#^ | 12.08±0.91^#^ |
| HGB/g·L^-1^ | 155.80±15.53 | 153.87±8.99 | 176.33±18.49^#^ | 183.67±13.09^#^ | 167.67±9.67^#^ | 173.21±10.71 |
| HCTL/L | 0.47±0.04 | 0.32±0.01 | 0.53±0.05^#^ | 0.57±0.04 | 0.53±0.03^#^ | 0.55±0.04 |
| MCV/fL | 51.75±0.55 | 51.56±0.58 | 48.27±0.54^#^ | 47.49±0.51^*#^ | 47.09±0.35^#^ | 46.19±0.69^*#^ |
| MCH/pg | 16.86±0.31 | 16.58±0.24^*^ | 15.83±0.28^#^ | 15.28±0.30^*#^ | 14.88±0.31^#^ | 14.35±0.65^*#^ |
| MCHC/g·L^-1^ | 325.80±8.23 | 321.50±3.88 | 328.10±4.74 | 321.70±4.51^*^ | 320.33±19.21 | 290.66±80.81 |
| PLT/10^9^·L^-1^ | 1179.70±453.02 | 1365.30±204.66 | 1422.50±105.43 | 1658.90±153.49 | 1319.47±86.55 | 1407.09±244.56 |
| LYM#/10^9^·L^-1^ | 1.23±0.54 | 1.11±0.52 | 0.74±0.36^#^ | 0.82±0.17^#^ | 1.00±0.24^#^ | 0.98±0.29^#^ |
| RDW-SD/fL | 40.39±2.80 | 41.80±1.44 | 27.70±0.36 | 27.92±0.32 | 26.77±0.34 | 27.91±0.36 |
| RDW-CV/% | 0.25±0.02 | 0.26±0.02 | 0.18±0.01^#^ | 0.19±0.01^*#^ | 0.17±0.01^#^ | 0.20±0.01^*#^ |
| PDW/fL | 6.49±0.16 | 6.53±0.10 | 6.83±0.17^#^ | 6.80±0.14^#^ | 6.84±0.18^#^ | 6.78±0.19^#^ |
| MPV/fL | 5.59±0.09 | 5.60±0.12 | 5.85±0.14^#^ | 5.84±0.10^#^ | 5.85±0.10^#^ | 5.76±0.10^*#^ |
| P-LCR/% | 0.02±0.01 | 0.02±0.01 | 0.03±0.01^#^ | 0.03±0.01^#^ | 0.03±0.00^#^ | 0.02±0.00^*^ |

* comparison of different sexes with the same age, p<0.05

# comparison to week 4 mice with the same gender, p<0.05

Table 4 Physiological indexes of NOD/SCID mice

| Indexes | Week 4 | | Week 8 | | Week 16 | |
| --- | --- | --- | --- | --- | --- | --- |
|  | ♀（*n*=10） | ♂（*n*=10） | ♀（*n*=10） | ♂（*n*=10） | ♀（*n*=10） | ♂（*n*=10） |
| WBC/10^9^·L^-1^ | 1.55±0.65 | 1.90±0.59 | 1.87±0.90 | 2.11±0.93 | 2.16±0.87^#^ | 2.23±0.85 |
| RBC/10^12^·L^-1^ | 5.93±0.96 | 6.79±1.50 | 4.76±1.12^#^ | 3.96±0.90^*#^ | 6.31±1.00 | 6.99±0.73^*^ |
| HGB/g·L^-1^ | 119.40±17.52 | 126.80±22.54 | 82.33±14.42^#^ | 71.47±24.80^#^ | 113.53±18.02 | 124.73±20.97 |
| HCT/% | 36.56±5.40 | 44.13±9.60^*^ | 30.44±6.28^#^ | 25.63±8.22^#^ | 38.54±7.44 | 40.01±7.69 |
| MCV/fL | 60.59±0.88 | 60.71±1.13 | 60.83±0.87 | 58.97±0.71^*#^ | 57.61±1.29^#^ | 56.67±0.70^*#^ |
| MCH/pg | 20.48±1.63 | 19.69±1.31 | 17.64±1.78^#^ | 15.85±2.06^*#^ | 18.04±0.82^#^ | 17.48±0.80^#^ |
| MCHC/g·L^-1^ | 333.20±25.34 | 327.13±18.48 | 290.00±29.78^#^ | 259.80±66.41^#^ | 295.13±74.51^#^ | 308.27±15.87^#^ |
| PLT/10^9^·L^-1^ | 759.80±188.20 | 792.87±173.37 | 417.20±135.46^#^ | 439.80±188.06^#^ | 786.93±226.66 | 741.87±192.31 |
| LYM#/10^9^·L^-1^ | 0.96±0.19 | 0.84±0.41 | 0.73±0.37^#^ | 0.69±0.36^#^ | 1.07±0.61 | 0.79±0.47 |
| RDW-SD/fL | 33.23±2.40 | 31.87±1.36 | 28.59±0.45^#^ | 27.43±0.62^*#^ | 31.11±1.32^#^ | 30.29±1.10^#^ |
| RDW-CV/% | 0.22±0.03 | 0.25±0.15 | 0.20±0.02 | 0.22±0.04 | 0.19±0.03 | 0.19±0.03 |
| PDW/fL | 6.47±0.31 | 6.70±0.48 | 7.31±1.01 | 6.73±0.52 | 6.61±0.34 | 6.66±0.30 |
| MPV/fL | 6.03±0.17 | 6.05±0.31 | 6.87±0.90 | 6.44±0.42 | 6.04±0.34 | 5.87±0.31 |
| P-LCR/% | 0.06±0.01 | 0.05±0.02 | 0.06±0.02 | 0.08±0.03 | 0.06±0.02 | 0.04±0.02 |

* comparison of different sexes with the same age, p<0.05

# comparison to week 4 mice with the same gender, p<0.05

Table 5 Biochemical indicators of SCID mice

| Indexes | Week 4 | | Week 8 | | Week 16 | |
| --- | --- | --- | --- | --- | --- | --- |
|  | ♀（*n*=10） | ♂（*n*=10） | ♀（*n*=10） | ♂（*n*=10） | ♀（*n*=10） | ♂（*n*=10） |
| TCHO/mmol·L^-1^ | 2.85±0.17 | 2.97±0.28 | 2.73±0.19 | 3.04±0.23^*^ | 2.59±0.26^#^ | 2.88±0.18^*^ |
| TG/mmol·L^-1^ | 1.19±0.23 | 1.40±0.34 | 1.15±0.31 | 1.34±0.36 | 0.65±0.10^#^ | 0.90±0.23^*#^ |
| HDL-C/mmol·L^-1^ | 1.88±0.11 | 2.00±0.19^*^ | 1.96±0.15 | 2.28±0.19^*#^ | 1.93±0.22 | 2.26±0.19^#^ |
| LDL-C/mmol·L^-1^ | 0.43±0.06 | 0.40±0.13 | 0.28±0.04^#^ | 0.22±0.03^*#^ | 0.28±0.05^#^ | 0.20±0.05^*#^ |
| TP/g·L^-1^ | 47.54±2.88 | 49.32±5.53 | 51.61±2.94^#^ | 51.79±2.09 | 53.49±5.77^#^ | 52.03±1.40 |
| ALB/g·L^-^ | 30.91±2.04 | 30.13±1.58 | 33.29±1.77^#^ | 28.95±1.05^*#^ | 29.46±7.63 | 28.65±1.92^#^ |
| ALT/IU·L^-^ | 42.33±7.25 | 45.20±10.43 | 28.67±5.02^#^ | 47.53±13.96^*^ | 35.80±10.93 | 43.6±19.33 |
| AST/IU·L^-^ | 132.53±29.69 | 133.93±44.71 | 85.13±9.89^#^ | 110.07±23.00^*^ | 119.2±26.53 | 112.80±19.68 |
| ALP/IU·L^-^ | 493.33±62.11 | 432.53±36.16^*^ | 284.80±24.36^#^ | 229.33±20.83^*#^ | 198.00±8.90^#^ | 180.87±15.26^*#^ |
| GLU/mmol·L^-1^ | 7.85±0.90 | 8.64±1.09^*^ | 7.09±0.71^#^ | 8.08±0.82^*#^ | 8.24±0.58 | 9.16±0.81^*^ |
| BUN/mmol·L^-1^ | 8.13±1.60 | 8.66±2.73 | 10.59±2.00^#^ | 13.04±4.93^#^ | 8.73±0.74 | 10.53±3.35 |
| CRE/μmoI/L | 14.93±3.08 | 17.07±4.96 | 17.40±5.49 | 20.33±18.40 | 23.60±1.80^#^ | 27.33±15.78 |
| Ca/mmol·L^-1^ | 2.28±0.07 | 2.20±0.10^*^ | 2.17±0.05^#^ | 2.10±0.03^*#^ | 2.06±0.52^#^ | 2.01±0.05^#^ |
| P/mmol·L^-1^ | 3.50±0.36 | 3.36±0.34 | 2.80±0.28^#^ | 2.92±0.47^#^ | 2.34±0.18^#^ | 2.24±0.30^#^ |

* comparison of different sexes with the same age, p<0.05

# comparison to week 4 mice with the same gender, p<0.05

Table 6 Biochemical indicators of NOD/SCID mice

| Indexes | Week 4 | | Week 8 | | Week 16 | |
| --- | --- | --- | --- | --- | --- | --- |
|  | ♀（*n*=10） | ♂（*n*=10） | ♀（*n*=10） | ♂（*n*=10） | ♀（*n*=10） | ♂（*n*=10） |
| TCHO/mmol·L^-1^ | 2.24±0.15 | 2.31±0.16^*^ | 2.69±0.23 | 2.80±0.33^#^ | 2.59±0.26 | 2.60±0.33 |
| TG/mmol·L^-1^ | 0.41±0.09 | 0.32±0.11^*^ | 0.63±0.15^#^ | 0.73±0.19^#^ | 0.37±0.09 | 0.31±0.10 |
| HDL-C/mmol·L^-1^ | 0.49±0.12 | 0.48±0.17^*^ | 0.64±0.13^#^ | 0.84±0.14^*#^ | 0.70±0.17^#^ | 0.84±0.34^#^ |
| LDL-C/mmol·L^-1^ | 0.46±0.05 | 0.35±0.05 | 0.31±0.06^#^ | 0.15±0.03^*#^ | 0.44±0.11 | 0.24±0.04^*#^ |
| TP/g·L^-1^ | 14.36±2.70 | 18.04±3.14^*^ | 46.75±3.99^#^ | 41.52±3.42^*#^ | 20.63±4.74^#^ | 18.89±5.58 |
| ALB/g·L^-^ | 31.65±2.43 | 28.53±3.81^*^ | 34.58±2.49 | 28.53±2.53^*^ | 34.74±4.69 | 30.89±5.64 |
| ALT/IU·L^-^ | 13.67±2.66 | 18.33±6.06^*^ | 34.17±43.40 | 16.92±5.88 | 26.93±5.69 | 25.60±6.59^#^ |
| AST/IU·L^-^ | 110.60±14.27 | 128.07±25.36^*^ | 151.92±81.74 | 93.75±13.29^*#^ | 135.40±26.23 | 108.27±21.87^*#^ |
| ALP/IU·L^-^ | 243.67±29.85 | 184.48±31.65^*^ | 147.17±14.64^#^ | 90.75±14.10^*#^ | 91.67±18.81^#^ | 80.87±8.38^*#^ |
| GLU/mmol·L^-1^ | 4.91±0.63 | 2.85±0.66^*^ | 5.04±0.78^#^ | 5.75±0.71^*#^ | 4.73±0.60^#^ | 5.09±0.79^#^ |
| BUN/mmol·L^-1^ | 6.47±0.66 | 13.54±5.64^*^ | 8.13±0.62 | 8.14±0.69^#^ | 7.58±0.78 | 8.72±1.00^*#^ |
| CREA/μmoI/L | 17.93±5.47 | 15.80±4.21 | 17.67±3.28 | 13.33±1.40^*^ | 17.93±2.02 | 13.57±1.75^*^ |
| Ca/mmol·L^-1^ | 2.08±0.16 | 1.78±0.20^*^ | 1.66±0.09^#^ | 1.53±0.09^*#^ | 1.92±0.23^#^ | 1.79±0.29 |
| P/mmol·L^-1^ | 4.17±0.36 | 4.34±0.69 | 3.36±0.37^#^ | 3.06±0.26^*#^ | 3.04±0.38^#^ | 3.02±0.36^#^ |

* comparison of different sexes with the same age, p<0.05

# comparison to week 4 mice with the same gender, p<0.05

Table 7 Immunoglobulins and complements of SCID mice

| Indexes | Week 4 | | Week 8 | | Week 16 | |
| --- | --- | --- | --- | --- | --- | --- |
|  | ♀（*n*=10） | ♂（*n*=10） | ♀（*n*=10） | ♂（*n*=10） | ♀（*n*=10） | ♂（*n*=10） |
| IgG（g/L） | 0.05±0.02 | 0.09±0.07 | 0.02±0.01^#^ | 0.02±0.01^#^ | 0.01±0.01^#^ | 0.02±0.01^*#^ |
| IgA（g/L） | 0.19±0.03 | 0.23±0.08^*^ | 0.11±0.01^#^ | 0.11±0.02^#^ | 0.10±0.02^#^ | 0.12±0.01^*#^ |
| IgM（g/L） | 0.07±0.02 | 0.09±0.05 | 0.06±0.01^#^ | 0.06±0.01^#^ | 0.05±0.01^#^ | 0.06±0.01^*#^ |
| C3（10^-2^g/L） | 0.46±0.41 | 0.34±0.30 | 0.22±0.03^#^ | 0.23±0.06 | 0.12±0.02^#^ | 0.14±0.02^*^ |
| C4（10^-2^g/L） | 0.03±0.01 | 0.04±0.03 | 0.02±0.01^#^ | 0.03±0.01 | 0.02±0.00^#^ | 0.03±0.01^*^ |

* comparison of different sexes with the same age, p<0.05

# comparison to week 4 mice with the same gender, p<0.05

Table 8 Immunoglobulins and complements of NOD/SCID mice

| Indexes | Week 4 | | Week 8 | | Week 16 | |
| --- | --- | --- | --- | --- | --- | --- |
|  | ♀（*n*=10） | ♂（*n*=10） | ♀（*n*=10） | ♂（*n*=10） | ♀（*n*=10） | ♂（*n*=10） |
| IgG（g/L） | 0.11±0.05 | 0.10±0.06 | 0.02±0.02^#^ | 0.04±0.04^#^ | 0.09±0.01 | 0.09±0.04 |
| IgA（g/L） | 0.04±0.03 | 0.05±0.04 | 0.06±0.02^#^ | 0.07±0.02^#^ | 0.03±0.02 | 0.03±0.03 |
| IgM（g/L） | 0.05±0.02 | 0.08±0.03^*^ | 0.07±0.01 | 0.05±0.01 | 0.05±0.03 | 0.05±0.02 |
| C3（10^-2^g/L） | 0.39±0.04 | 0.41±0.03 | 0.46±0.03^#^ | 0.36±0.03^*#^ | 0.46±0.04^#^ | 0.36±0.05^*#^ |
| C4（10^-2^g/L） | 0.01±0.00 | 0.01±0.00 | 0.02±0.00^#^ | 0.02±0.00^#^ | 0.00±0.00^#^ | 0.00±0.00^#^ |

* comparison of different sexes with the same age, p<0.05

# comparison to week 4 mice with the same gender, p<0.05

### Immunol cells collection and measurements

Methods

20 8-week mice with half females and half males was euthanized to take the spleen. Adipose tissue of each mice was removed as far as possible, and each of the tissue was put into the lymphocyte separation fluid to be fully grinded. The separation fluid was transferred to 15mL centrifugal tubes, covered with 200-500μL RPMI-1640 medium. Centrifugate at 800 x g for 30 min at 4°C (Eppendorf 5427R centrifuge, Hamburg, Germany) to extract the layer of lymphocytes, then add 10mL RPMI-1640 medium, and centrifugate at 250 x g for 10min to remove the supernatant. Flow cytometry analysis (BD Bioscience, US) was then conducted to count T cells and their subsets, B cells (B220+) and NK cells (NK1.1+).

Results

Table 9 Immune cell composition of SCID mice (%)

| Gender | T cells | | | | | B220+ | NK cells |
| --- | --- | --- | --- | --- | --- | --- | --- |
|  | CD3+ | CD4+CD8- | CD4-CD8+ | CD4+CD8+ | CD4-CD8- |  |  |
| ♀（*n*=10） | 0.36±0.36 | 2.45±1.78 | 9.70±7.48 | 6.27±3.41 | 77.57±12.48 | 51.42±16.06 | 16.35±2.16 |
| ♂（*n*=10） | 0.38±0.14 | 2.50±1.04 | 12.53±5.65 | 7.08±4.81 | 47.64±29.94 | 43.28±25.00 | 16.08±6.59 |

Table 10 Immune cell composition of NOD/SCID mice (%)

| Gender | T cells | | | | | B220+ | NK cells |
| --- | --- | --- | --- | --- | --- | --- | --- |
|  | CD3+ | CD4+CD8- | CD4-CD8+ | CD4+CD8+ | CD4-CD8- |  |  |
| ♀（*n*=10） | 0.35±0.15 | 36.33±21.50 | 13.36±4.90 | 12.82±11.45 | 37.50±16.65 | 26.21±7.66 | 10.84±3.71 |
| ♂（*n*=10） | 1.03±0.93 | 41.34±35.99 | 11.25±14.01 | 12.20±16.60 | 35.21±7.35 | 21.56±9.37 | 10.42±1.74 |
