# Supplementary material for: Immunodeficiency Promotes Adaptive Alterations of Host Gut Microbiome: An Observational Metagenomic Study in Mice
Source: Front Microbiol. 2019 Nov 1;10:2415. doi: 10.3389/fmicb.2019.02415 (PMC6853035; doi:10.3389/fmicb.2019.02415)
Supplement: Supplementary file 3 [file Image_1.pdf]

Supplemental Figures S1-3 for "Immunodeficiency promotes adaptive alterations of host gut microbiome: An observational metagenomic study in mice"

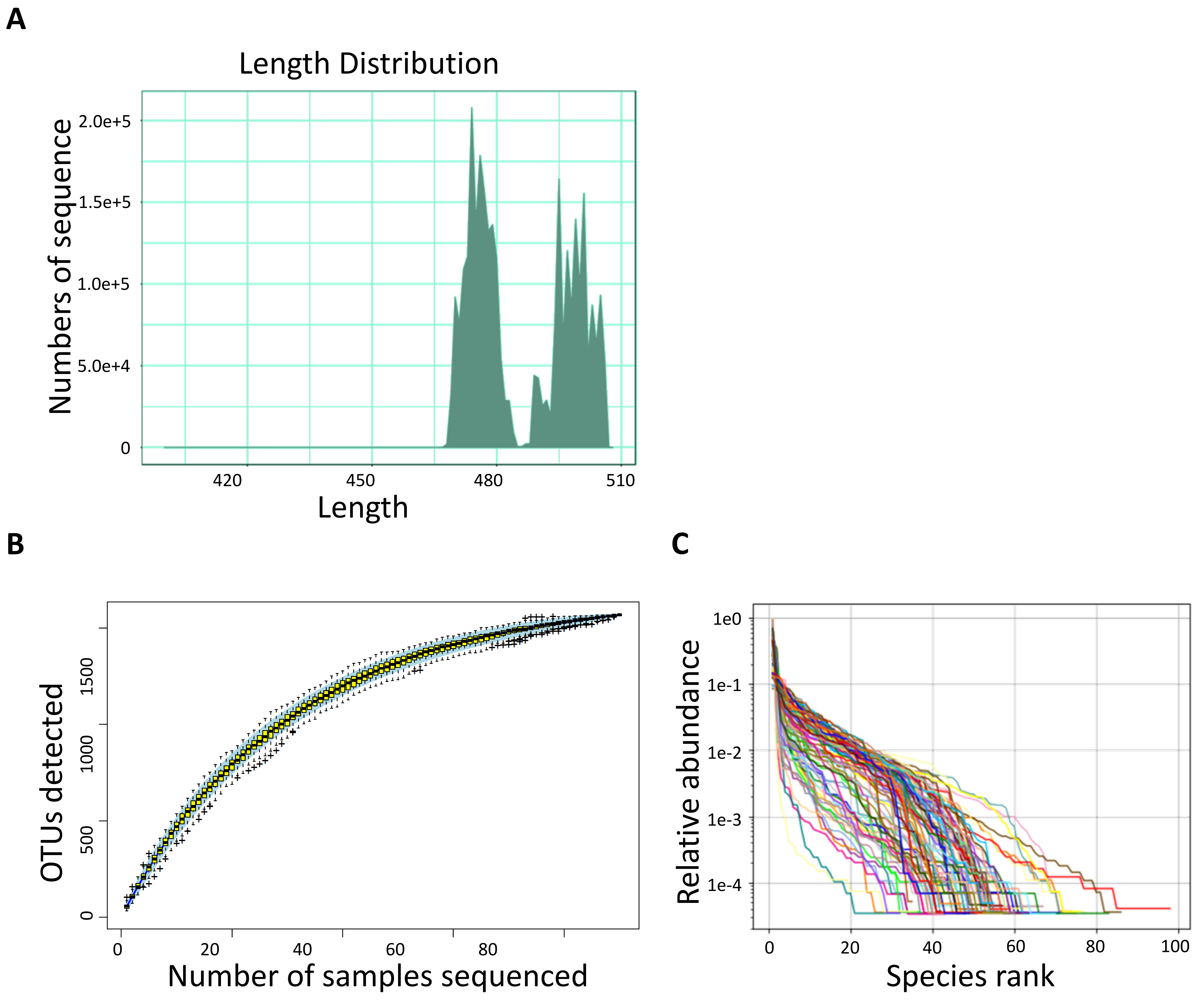

Figure S1 (A) The length distribution of sequence reads. (B) Species accumulation curves. (c) Rarefaction curves (at a 97% similarity level) of fecal microbiota.

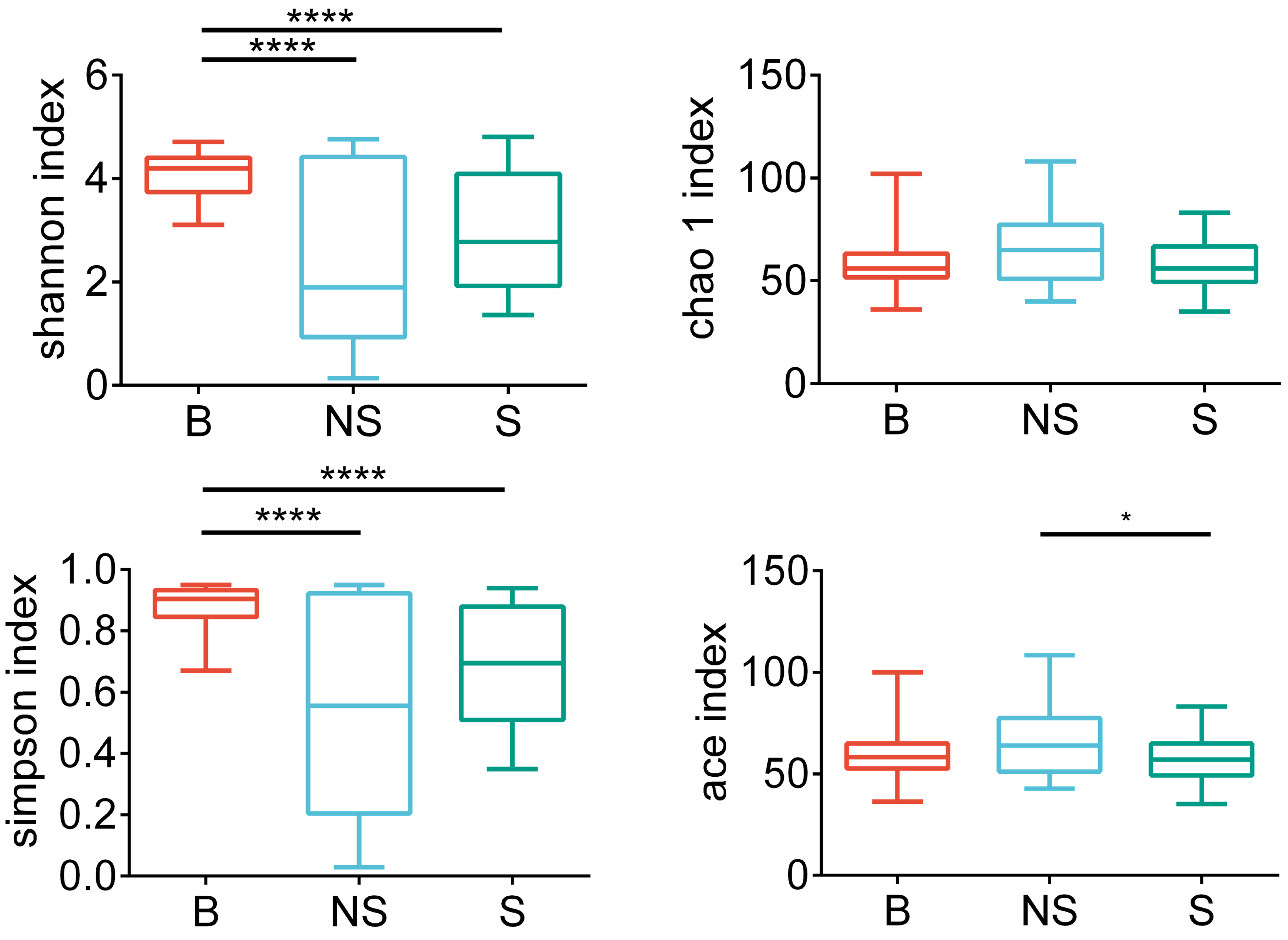

Figure S2 Alpha diversity of three groups' fecal microbiota. Three alpha diversity index chao1 index, Shannon index, Simpson index and ace index in BALB/CA mice group SCID mice group and NOD/SCID mice group.

B: BALB/CA mice group S:SCID mice group NS: NOD/SCID mice group. $p<0.05$*. $p<0.01$**. $p<0.001$*** $p<0.0001$****

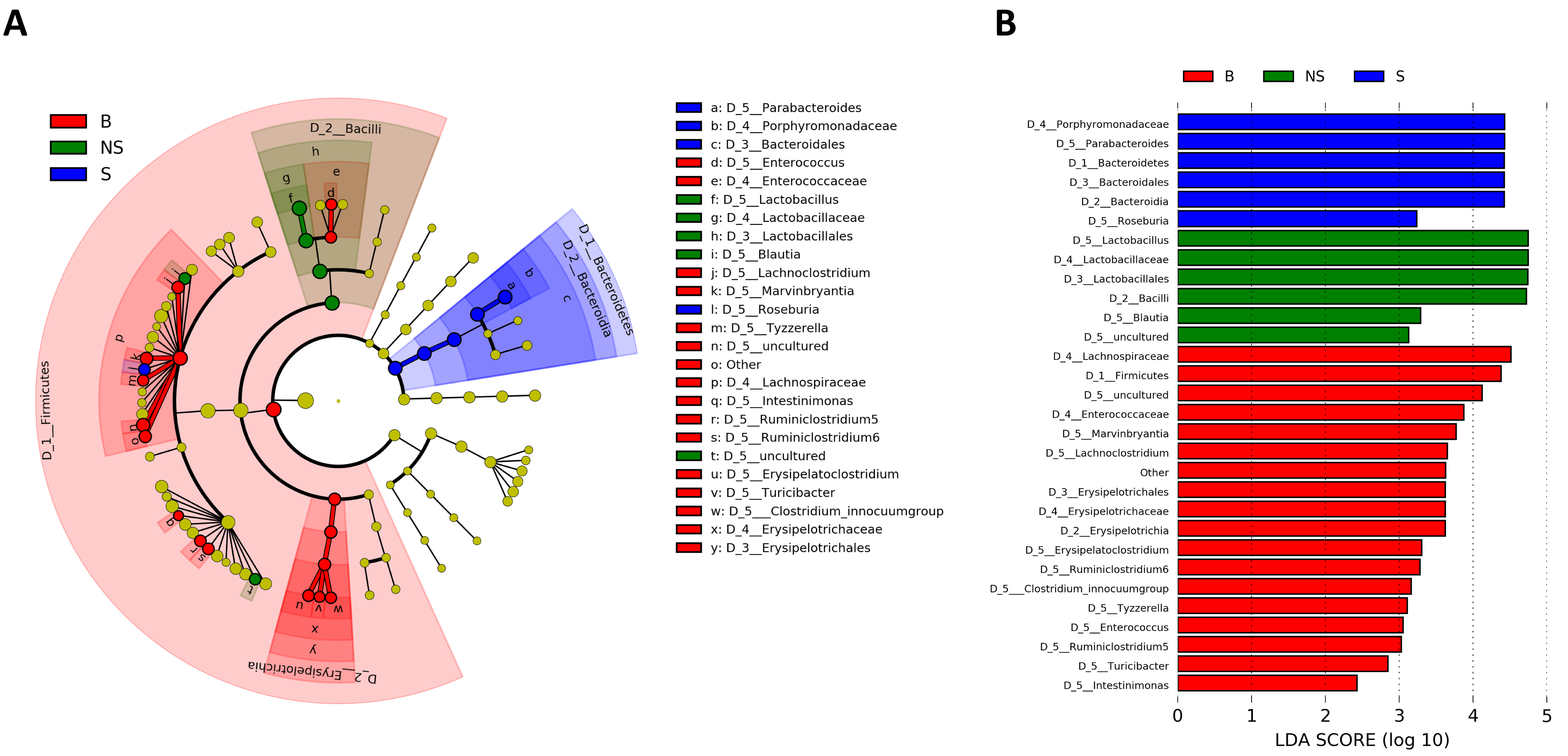

Figure S3 Different structures of the microbiota in three mice lines. (A) Taxonomic representation of statistically and biologically consistent differences between BALB/CA and SCID mice. Differences are represented by the color of the most abundant class (red indicates BALB/CA mice, green indicates NOD/SCID mice, and blue indicates SCID mice). The diameter of each circle is proportional to the taxon's abundance. (B) Histogram of the LDA scores for differentially abundant genera. The cladogram was calculated by LEfSe, a metagenome analysis of abundant taxons of OTUs; the cladogram is displayed according to the effect size. Only taxa meeting an LDA significant threshold > 2 and $p < 0.05$ are shown.
